# Supplementary material for: Transient inhibition of cell division in competent pneumococcal cells results from deceleration of the septal peptidoglycan complex
Source: Nat Commun. 2025 Jul 1;16:5666. doi: 10.1038/s41467-025-60600-z (PMC12214887; doi:10.1038/s41467-025-60600-z)
Supplement: Supplementary file 1 — Supplementary Information [file 41467_2025_60600_MOESM1_ESM.pdf]

Supplementary Information

Transient inhibition of cell division in competent pneumococcal cells results from deceleration of the septal peptidoglycan complex

Dimitri Juillot[1,2,3], Cyrille Billaudeau[1], Isabelle Mortier-Barrière[2,3], Aurélien Barbotin[1], Armand Lablaine[1], Patrice Polard[2,3], Nathalie Campo[2,3]✉ and Rut Carballido-López[1]✉

[1]Université Paris-Saclay, INRAE, AgroParisTech, Micalis Institute, 78350, Jouy-en-Josas, France

[2]Laboratoire de Microbiologie et Génétique Moléculaires, UMR5100, Centre de Biologie Intégrative, Centre Nationale de la Recherche Scientifique, 31062 Toulouse, France.

[3]Université Paul Sabatier (Toulouse III), 31062 Toulouse, France.

These authors contributed equally: Cyrille Billaudeau and Isabelle Mortier-Barrière

✉ Corresponding authors.

E-mail: nathalie.campo[at]univ-tlse3.fr, rut.carballido-lopez[at]inrae.fr

Supplementary Information:

Supplementary Tables 1: *Average speed of proteins analyzed in this study.*

Supplementary Tables 2: *Strains used in this study.*

Supplementary Tables 3: *Primers used in this study.*

Supplementary Figures S1 to S14

Supplementary References

Supplementary Table 1. *Average speed of proteins analyzed in this study.*

**Protein**	**Figure**	**non-competent cells[a]**	**competent cells[a]**	**non-competent cells + methicillin[a]**	**competent cells + methicillin[a]**	**competent cells ΔcomM background[a]**	**non-competent cells + comM expression[a]**	**non-competent cells + divIB over-expression[a]**	**competent cells + divIB over-expression[a]**
mNeonGreen-ComM	Fig. 1h	n.a.	12.2 ± 5.3						
	Fig. 3f		14.6 ± 5.5		9.5 ± 7.5				
FtsZ-mNeonGreen	Fig. 2c	32.9 ± 11.2	33.3 ± 10.9						
	Sup Fig. 9	30.8 ± 8.5		30.9 ± 8.8					
mNeonGreen-PBP2x	Fig. 3b	21.0 ± 5.8	11.8 ± 5.4						
	Fig. 3e		15.1 ± 7.3		10.1 ± 7.1				
	Sup Fig. 9	22.6 ± 7.7		12.7 ± 7.9					
	Fig. 5a		16.1 ± 5.9			20.8 ± 7.7			
	Fig. 5b	21.2 ± 6.2					17.4 ± 6.5		
	Fig. 7d		14.9 ± 7.2						17.4 ± 7.5
	Sup Fig. 11	22.3 ± 6.4						21.7 ± 6.4	
FtsW-mNeonGreen	Fig. 3b	20.2 ± 6.9	14.2 ± 6.6						
GFP-PBP2b	Fig. 3b	20.9 ± 6.9	21.1 ± 6.9						
RodA-mNeonGreen	Fig. 3b	22.5 ± 8.8	21.7 ± 8.3						
mNeonGreen-DivIB	Fig. 7a	22.3 ± 6.0	14.9 ± 5.4						

[a] *speed measured in nm/s.*

Supplementary Table 2. *Strains used in this study.*

Strain	Genotype/relevant features[a]	Source/Reference
R1501	Δ*comC*	1
R4599	Δ*comC*, *ftsZ-mNeonGreen*	This study
R4601	Δ*comC*, *mNeonGreen-comM*	This study
R4728	Δ*comC*, *ftsW-mNeonGreen*	This study
R4743	Δ*comC*, *mNeonGreen-pbp2x*	This study
R4744	Δ*comC*, *HaloTag-pbp2x*	This study
R4746	Δ*comC*, *mNeonGreen-comM, HaloTag-pbp2x*	This study
R4858	Δ*comC*, *dprA-LgBit,* CEP_{PLac}-*dprA-SmBit* (*kan*); Kan^R	2
R4845	Δ*comC*, *mNeonGreen-pbp2x, comM::aad9;* $Spec^R$	This study
R4846	Δ*comC*, *mNeonGreen-pbp2x,* CEP_R-*comM* (*kan*); Kan^R	This study
R4867	Δ*comC*, *rodA-mNeonGreen*	This study
R4869	Δ*comC*, *mNeonGreen-divIB*	This study
R5224	Δ*comC,* CEP_{PLac}-*divIB* (*kan*); Kan^R	This study
R5225	Δ*comC*, *mNeonGreen-pbp2x,* CEP_{PLac}-*divIB* (*kan*); Kan^R	This study
R5276	Δ*comC*, *LgBiT-comM*	This study
R5285	Δ*comC*, *LgBiT-comM, SmBiT-divIB*	This study
R5292	Δ*comC*, *LgBiT-comM,* CEP_{PLac}-*dprA-SmBit* (*kan*); Kan^R	This study
R5297	Δ*comC*, *mNeonGreen-comM, rodA-HaloTag*	This study
R5298	Δ*comC*, *ALFA-comM*	This study
R5300	Δ*comC*, *mNeonGreen-divIB*, *ALFA-comM*	This study
R5311	Δ*comC*, *FtsW-SmBiT*	This study
R5312	Δ*comC*, *LgBiT-comM, FtsW-SmBiT*	This study
R5313	Δ*comC*, *LgBiT-comM,* CEP_{PLac}-*dprA-SmBit* (*kan*), *dprA*::*spc*; Kan^R, Spc^R	This study
R5315	Δ*comC*, *LgBiT-comM, SmBiT-pbp2x*	This study
R5317	Δ*comC*, *FtsW-SmBiT, LgBiT-pbp2x*	This study
R5384	Δ*comC*, *mNeonGreen-divIB*, *comM::aad9*, *cbpD::cat*; $Spec^R$, Cam^R	This study
R5385	Δ*comC*, *ftsW-mNeonGreen, ALFA-comM*	This study
R5386	Δ*comC*, *ftsW-mNeonGreen, HaloTag-pbp2x*	This study
R5389	Δ*comC*, *mNeonGreen-comM, HaloTag-divIB*	This study
WT gfp-pbp2b	*rpsL1*, *gfp-pbp2b*; Str^R	3

a: ^{R}, resistance; Kan, kanamycin; Spec, spectinomycin; Str, streptomycin; Cam, chloramphenicol

Supplementary Table 3. *Primers used in this study*

Primer	Sequence
OEC48	GGTCAAACGACTGTCGCTACAA
OEC49	CCAGTTCTCGATACTGTTCTAGCTT
OEC88	GACCTATCTCTTCATCGCCCAT
OEC111	CCACCAGCATCCGCAATCA
DJ11	GATTGGTAGGAAGGGAGAGAGAAGATGGTCTCTAAAGGTGAAGAAGATAATATGGCT
DJ12	GCTAACAAAAATAAGATTCTCATTGATTTTCCGGAACCCTCGAGTTTATACAATTCATCC ATACCCATTACA
DJ13	TATTATCTTCTTCACCTTTAGAGACCATCTTCTCTCTCCCTTCCTACCAATC
DJ14	TCTGTGCCACCATAAACCAATTGC
DJ15	AATGGGTATGGATGAATTGTATAAACTCGAGGGTTCCGGAAAATCAATGAGAATCTTAT TTTTGTTAGC
DJ16	CCTCTCATATTGACCATCACATAGCG
DJ47	TTTAGCTGCTGCTTCTCCACCAGATCCTGAACGATTTTTGAAAAATGGAGGTGTATC
DJ45	TCTGGTGGAGAAGCAGCAGCTAAAGCTGGAGTCTCTAAAGGTGAAGAAGATAATATGG CT
DJ46	CAAGTTCTGTATTTTCTTTTACATTCATTTACTTATTTATACAATTCATCCATACCCATTAC ATCA
DJ48	CTGATGTAATGGGTATGGATGAATTGTATAAATAAGTAAATGAATGTAAAAGAAAATACA GAACTTG
DJ59	GGAAGGCTTTGATGAGCTGGTTC
DJ60	GCTGCTTCTCCACCAGATCCTGATTTAATTTGTTTTAATACAACCTTTTTCCGTTTGAAT GGGA
DJ61	CGGAAAAAGGTTGTATTAAAACAAATTAAATCAGGATCTGGTGGAGAAGCAGCA
DJ62	GCTACTTTTACCATGATTTTCTCCTTATTTATACAATTCATCCATACCCATTACATCAG
DJ63	GTAATGGGTATGGATGAATTGTATAAATAAGGAGAAAATCATGGTAAAAGTAGCAGT
DJ64	CATGCGTTCCACCTTCATGTG
DJ79	GGTAAGACAACCACAACGACTATG
DJ80	CTTCACCTTTAGAGACCATTTACTTTCCTTATGATAAATCTTTTTTCAACAATTG
DJ81	GAAAAAAGATTTATCATAAGGAAAGTAAATGGTCTCTAAAGGTGAAGAAGATAATATG
DJ82	GTCCTCATTTTTCTTATCTTTTGATCCGGAACCCTCGAGTTTATAC
DJ83	GTATAAACTCGAGGGTTCCGGATCAAAAGATAAGAAAAATGAGGACAAAGAAAC
DJ84	GAGTCCAAGCAATTTATACGATGTG
mAD1ext	AACGGTCCGAACCTCATAACA

mBD2ext	AGCTTCTGAATAAGCCCTCG
OCN334	TTCCCCGGGGGATCCATGAAATCAATGAGAATCTTATTTTTGTTAG
OCN365	CAGGTCGACATCGATCTACTTCAACAGAAGGTTCATTGG
OCN366	TTCCCCGGGGGATCCATGAAGTGGACAAAAAGAGTAATCC
OCN367	CAGGTCGACATCGATTTAGTCTCCTAAAGTTAATGTAATTTTTTTAATG
OCN423	ATCGATGTCGACCTGCAGAGATC
OCN424	GGATCCCCCGGGGAATTC
OCN430	CAGGTCGACATCGATCCTACTAAACTTATCATCATAACATCCA
OCN440	GGCTCGCCATGATAAGAGCGA
OCN441	CCACCAGATCCTCTGGATCCGGCCTTCAACAGAAGGTTCATTGGTTG
OCN442	CAACCAATGAACCTTCTGTTGAAGGCCGGATCCAGAGGATCTGGTGGAGAAGC
OCN443	GACATAAACTATCCTTTCTTTATCCTATTATTTATACAATTCATCCATACCCATTACATCA
OCN444	GTATGGATGAATTGTATAAATAATAGGATAAAGAAAGGATAGTTTATGTC
OCN445	GCCTTGATTGGACTTGACTCGAAG
OCN450	GGCTGCGTTGAGGACAGGTATC
OCN451	CATATTATCTTCTTCACCTTTAGAGACCATATCTTACTCCGCTATTCTAATATTTTCA
OCN452	GAAAATATTAGAATAGCGGAGTAAGATATGGTCTCTAAAGGTGAAGAAGATAATATG
OCN453	CGGATTACTCTTTTTGTCCACTTCATTCCGGAACCCTCGAGTTTATAC
OCN454	GTATAAACTCGAGGGTTCCGGAATGAAGTGGACAAAAAGAGTAATCCGT
OCN455	CCAACCATACATATCTGGAACCTCCTC
OCN456	CAGTACCAATTTCAGCCATATCTTACTCCGCTATTCTAATATTTTCATTG
OCN457	GAAAATATTAGAATAGCGGAGTAAGATATGGCTGAAATTGGTACTGGT
OCN458	CGGATTACTCTTTTTGTCCACTTCATACCTGAACCTTGACCACTTCCTG
OCN459	GGAAGTGGTCAAGGTTCAGGTATGAAGTGGACAAAAAGAGTAATCCGT
OCN560	CTCTTCTTCTCTTGACTTTTCACTCAAATGTACACCTCCTTAAGCTTAATTGTTATCC
OCN561	CAATTAAGCTTAAGGAGGTGTACATATGTCAAAAGATAAGAAAAATGAGGACAAAGAAA CCCT
OCN562	CCATTAAAAATCAAACGGATCCCTAGCGACGCGATGAACGC
OCN563	GCGTTCATCGCGTCGCTAGGGATCCGTTTGATTTTTAATGGATAATGTG
OCN564	CCCTTGTCTTGGTATCCTTGTC
OCN606	CTTAGTTCCTCTTCAAGGCGACTAGGAGCTTTAGCTGCAGCTTCTCCAC

OCN607	CGCCTTGAAGAGGAACTAAGAAGGCGGCTAACAGAATGAGATAAAGAAAGGATAGTTTATGTCTC
OCN609	CGCCTTGAAGAGGAACTAAGAAGGCGGCTAACAGAACCACTCGAGGGTTCCGGAAAATC
OCN632	GAAAACCAGTACCAATTTCAGCCATTTACTTTCCTTATGATAAATCTTTTTTCAACAATTG
OCN633	CAATTGTTGAAAAAAGATTTATCATAAGGAAAGTAAATGGCTGAAATTGGTACTGGTTTTC
OCN634	CTTTGTCCTCATTTTTCTTATCTTTTGAACCTGAACCTTGACCACTTCC
OCN635	GTGGTCAAGGTTCAGGTTCAAAAGATAAGAAAAATGAGGACAAAGAAAC
OCN647	CCTTCTTAGTTCCTCTTCAAGGCGACTCATCTTCTCTCTCCCTTCCTACC
oIM149	CTTCTTGTTCAAATTTTCCC
oIM245	ATGGTTACAGGTTATAGACTTTTTGAAGAAATTCTACTCGAGGGTTCCGG
oIM282	CACAAAATCTTCAAGTGTAAAAACCATCTTCTCTCTCCCTTCCTACCAATC
oIM283	GATTGGTAGGAAGGGAGAGAGAAGATGGTTTTTACACTTGAAGATTTTGTGGG
oIM284	CAAAAATAAGATTCTCATTGATTTTCCGGAACCCTCGAGACTGTTTATAGTTACTC
oIM285	CTCGAGGGTTCCGGAAAATCAATGAGAATCTTATTTTTGTTAG
oIM286	TTCAAAAAGTCTATAACCTGTAACCATTTACTTTCCTTATGATAAATCTTTTTTCAACAATTG
oIM290	GAATTTCTTCAAAAAGTCTATAACCTGTAACAGCTTTAGCTGCAGCTTCTCC
oIM291	GGTTATAGACTTTTTGAAGAAATTCTATAAGATAAAGAAAGGATAGTTTATGTCTC
oIM292	TTCAAAAAGTCTATAACCTGTAACCATATCTTACTCCGCTATTCTAATATTTTCATTG
oIM293	CACAAAATCTTCAAGTGTAAAAACCATATCTTACTCCGCTATTCTAATATTTTCATTG
oIM294	ATATTAGAATAGCGGAGTAAGATATGGTTTTTACACTTGAAGATTTTGTGGG
oIM295	TCCGGAACCCTCGAGACTGTTTATAGTTACTCTAAACAACATAGATCC
oIM296	CTATGTTGTTTAGAGTAACTATAAACAGTCTCGAGGGTTCCGGA

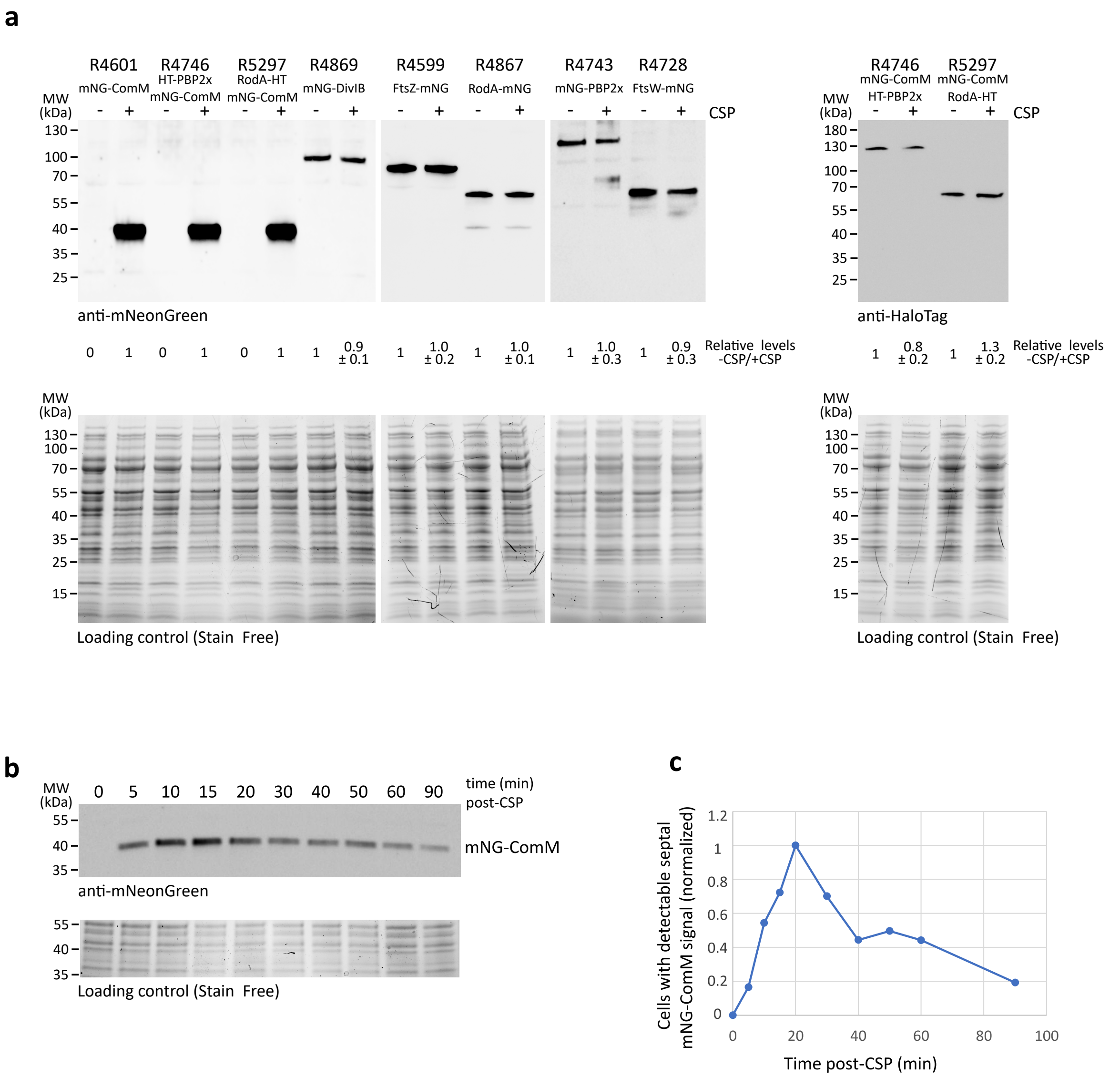

Supplementary Figure 1. The fluorescent protein fusions are expressed and not degraded.
a Western blots to detect the fluorescent protein fusions. Cells of the indicated strains were induced to develop competence for 15 min. Whole cell extracts were prepared and analyzed by immunoblot using anti-mNeonGreen or anti-HaloTag antibodies as indicated. Stain free images were used as loading controls. Expected size of fluorescent protein fusions: mNG, 28 kDa; mNG-ComM, 50 kDa; FtsZ-mNG, 72 kDa; mNG-PBP2x, 109 kDa; FtsW-mNG, 73 kDa; RodA-mNG, 74 kDa; mNG-DivIB, 72 kDa; HT-PBP2x, 117 kDa; RodA-HT, 81 kDa. Relative levels for competent versus non competent cultures are indicated. Values represents the mean ± s.d. for three to six biological replicates.
b Western blot detection of mNeonGreen-ComM (strain R4601) at different times after competence induction (post-CSP addition). Whole cell extracts were analyzed with anti-mNeonGreen antibodies. Stain free images were used as loading controls. Data representative of two independent experiments.
c Proportion of cells of strain R4601 containing a detectable mNeonGreen-ComM signal at the septum, at different times after competence induction (post-CSP).
Uncropped blots are provided in Source Data File.

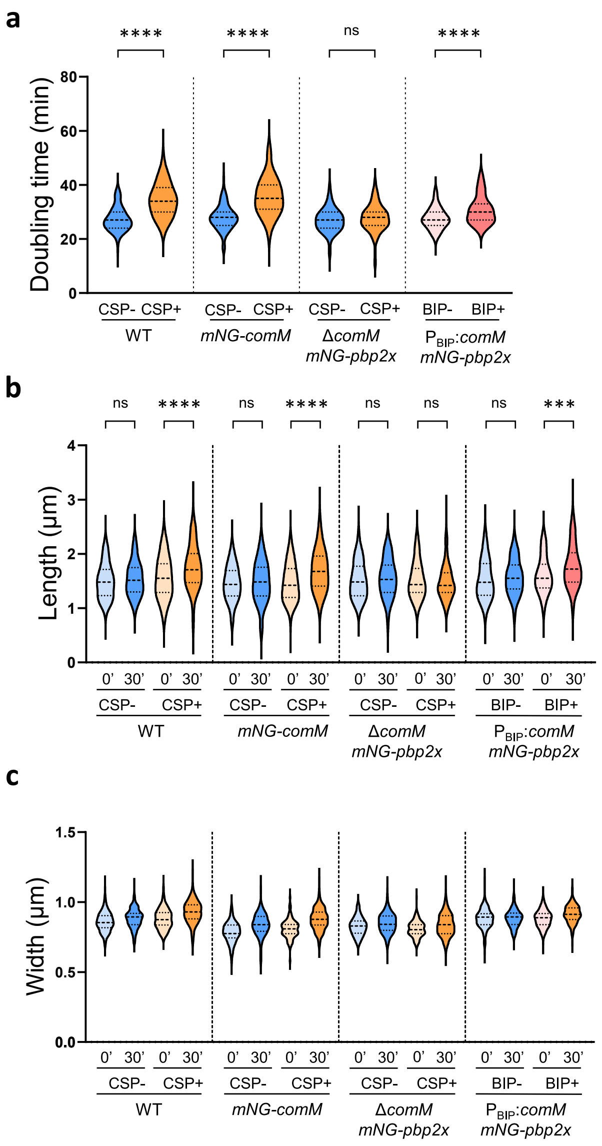

Supplementary Figure 2. Functionality of the ComM mutant strains used in this study.
Cells were grown in C+Y medium to early exponential phase and competence was induced (CSP+) or not (CSP-) by CSP addition. Strains analyzed are R1501 (wild-type), R4601 (mNeonGreen-ComM), R4845 (mNeonGreen-PBP2x, ΔcomM) and R4846 (mNeonGreen-PBP2x, PBIP-comM).
Data are presented as violin plots where the top and bottom dashed lines correspond to the 25th and 75th percentiles, and the middle-dashed line corresponds to the median. Pairwise comparisons were done with a nonparametric two-sided Mann-Whitney test. P values are displayed as follows: ****, $P < 0.0001$; ***, $0.0001 < P < 0.001$; ns, $P > 0.05$.
a Doubling time of single cells based on phase contrast time-lapses analyses (see Material and Methods). Number of cells analyzed over two independent biological replicates in non-competent and competent cultures are respectively 223 and 68 for R1501, 125 and 84 for R4601, and 134 and 129 for R4845. Number of cells analyzed over two independent biological replicates in R4846 cultures treated or not with BIP are respectively 110 and 159. P values were calculated for competent versus non competent cultures of strains R1501 (exact P<0.0001), R4601 (exact P<0.0001), R4845 (approximate P = 0.1049) and R4846 (approximate P<0.0001).
b,c Cell length (**b**) and cell width (**c**) distributions measured on phase contrast microscopy. Cells were imaged during 1 hour after incubation with or without CSP, or BIP, and their dimensions were measured at 0 min and 30 min. Number of cells analyzed at 0 min over two independent biological replicates in non-competent and competent cultures are respectively 166 and 166 for R1501, 130 and 135 for R4601, and 104 and 136 for R4845. Number of cells analyzed over two independent biological replicates in R4846 cultures treated or not with BIP are respectively 140 and 129. Number of cells analyzed at 30 min over two independent biological replicates in non-competent and competent cultures are respectively 274 and 239 for R1501, 257 and 146 for R4601, and 170 and 192 for R4845. Number of cells analyzed over two independent biological replicates in R4846 cultures treated or not with BIP are respectively 206 and 266. P values were calculated for lengths measured at 0 min and 30 min for non-competent R1501 (approximate P = 0.1349), competent R1501 (approximate P<0.0001), non-competent R4601 (approximate P = 0.4965), competent R4601 (approximate P<0.0001), non-competent R4845 (approximate P = 0.2681), competent R4845 (approximate P = 0.2844), non-competent R4846 (approximate P = 0.1092), and competent R4846 (approximate P = 0.0002).
Source data are provided as a Source data file.

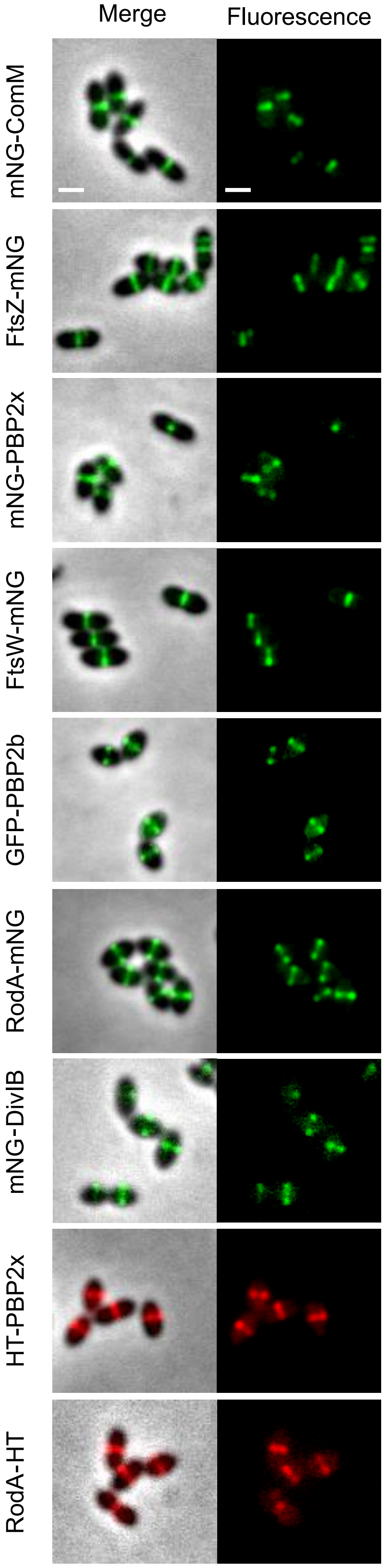

Supplementary Figure 3. ComM, FtsZ, PBP2x, FtsW, PBP2b, RodA and DivIB localize to the division ring at midcell.

Strains R4599 (FtsZ-mNG), R4743 (mNG-PBP2x), R4728 (FtsW-mNG), WT gfp-pbp2b (GFP-PBP2b), R4867 (RodA-mNG), R4869 (mNG-DivIB), R4744 (HT-PBP2x) and R5297 (mNG-ComM, RodA-HT) were grown in C+Y medium to OD_{550nm} ~ 0.1 (without CSP induction) and analyzed by epifluorescence microscopy. Strain R4601 (mNG-ComM) was induced to develop competence with CSP at OD_{550nm} ~ 0.1 for 15 minutes before imaging. Representative fluorescence (*Right*) and merged (*Left*) images of phase-

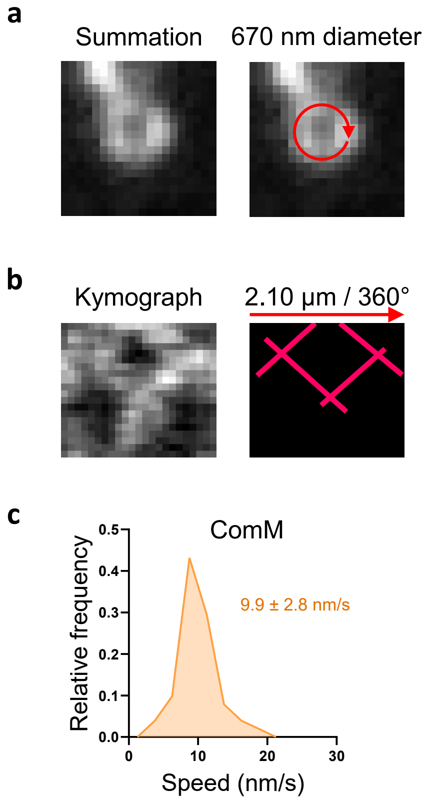

Supplementary Figure 4. ComM nodes move in both directions around the cell circumference.
Cells of strain R4601 were grown in C+Y medium to early exponential phase and induced to develop competence by CSP addition for 10 min before imaging. Cells were immobilized vertically and imaged by time-lapse HILO microscopy at 3 s intervals.

a Representative fluorescence intensity projection images. Summation of frames from 240 s HILO movie of mNeonGreen-ComM.

b Radial kymograph generated following the red line shown in a (*Left*) and cartoon version of the kymograph with multiple trajectories shown in red (*Right*).

c Distribution of speed of mNeonGreen-ComM in competent cells (n=51 trajectories measured over three independent replicates). Average speed ± sd is indicated. Source data are provided as a Source data file.

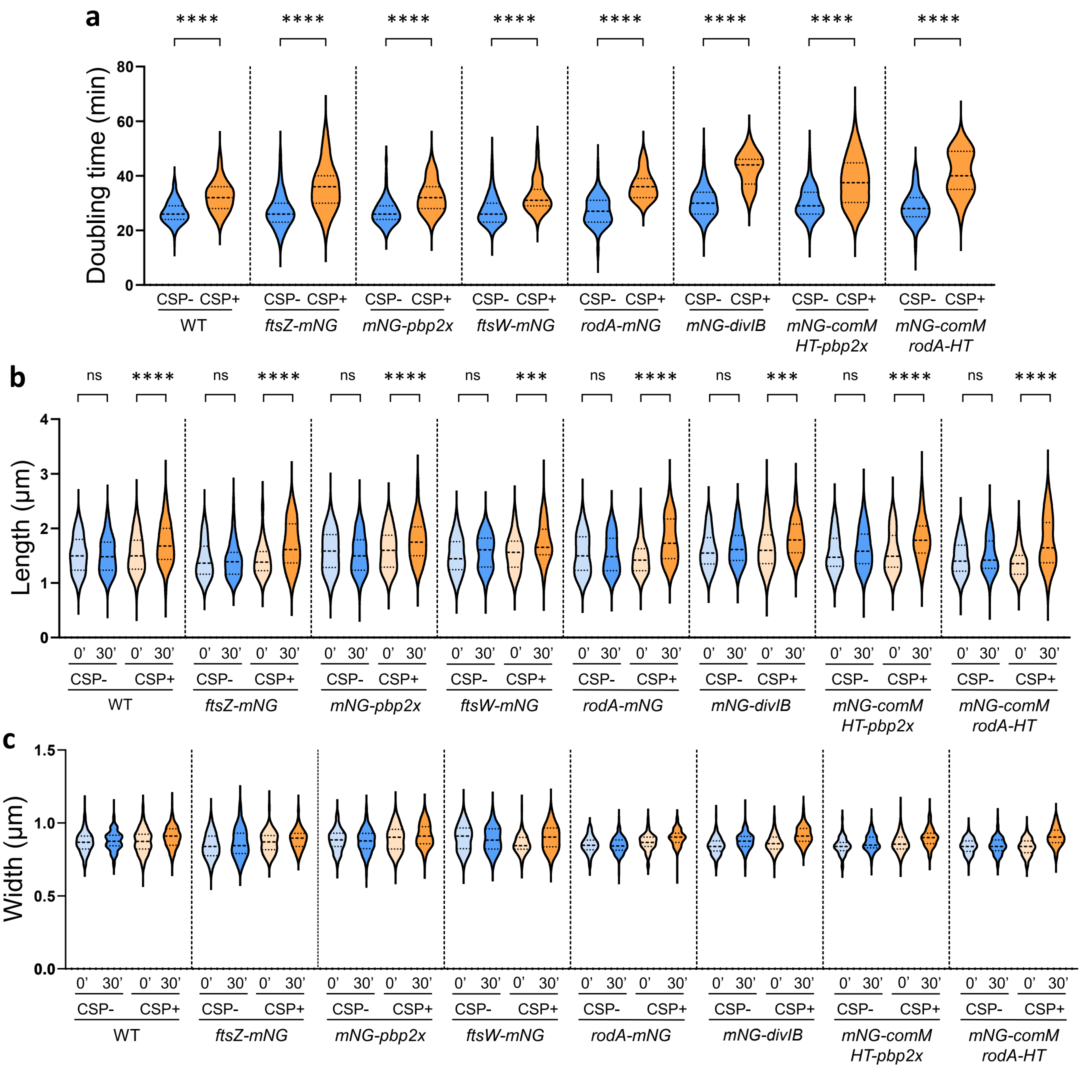

Supplementary Figure 5. Functionality of the fluorescent fusions used in this study.
Strains containing native mNeonGreen fusions of FtsZ (R4599), PBP2x (R4743), FtsW (R4728), RodA (4867), DivIB (R4869) as well as native mNeonGreen fusions of ComM combined with HaloTag fusion of PBP2x (4746) or RodA (5297), are expressed and have similar doubling time and cell dimensions compared to wild-type cells (R1501). Cells were grown in C+Y medium to early exponential phase and competence was induced (+) or not (-) by CSP addition. Data are presented as violin plots where the top and bottom dashed lines correspond to the 25th and 75th percentiles, and the middle-dashed line corresponds to the median. Pairwise comparisons were done with a nonparametric two-sidedMann-Whitney test. P values are displayed as follows: ****, $P < 0.0001$; ***, $0.0001 < P < 0.001$; ns, $P > 0.05$.
a Doubling time of single cells based on phase contrast time-lapses analyses. Number of cells analyzed over two independent biological replicates in non-competent and competent cultures are respectively 289 and 155 for R1501, 63 and 35 for R4599, 153 and 115 for R4743, 122 and 59 for R4728, 101 and 48 for R4867, 130 and 20 for R4869, 116 and 92 for R4746 and 104 and 59 for R5297. *P* values were calculated for competent versus non competent cultures of strains R1501 (approximate $P<0.0001$), R4599 (exact $P<0.0001$), R4743 (approximate $P<0.0001$), R4728 (exact $P<0.0001$), R4867 (exact $P<0.0001$), R4869 (exact $P<0.0001$), R4746 (exact $P<0.0001$) and R5297 (exact $P<0.0001$).
b,c Cell length (**b**) and cell width (**c**) distributions measured on phase contrast microscopy images. Cells were imaged during 1 hour after incubation with or without CSP. Dimensions were measured 0 min and 30 min post-CSP addition. Number of cells analyzed at 0 min over two independent biological replicates in non-competent and competent cultures are respectively 212 and 299 for R1501, 58 and 57 for R4599, 175 and 176 for R4743, 99 and 91 for R4728, 118 and 144 for R4867, 118 and 85 for R4869, 124 and 139 for R4746 and 119 and 107 for R5297. Number of cells analyzed at 30 min over two independent biological replicates in non-competent and competent cultures are respectively 469 and 475 for R1501, 123 and 81 for R4599, 394 and 274 for R4743, 187 and 133 for R4728, 245 and 201 for R4867, 221 and 90 for R4869, 232 and 214 for R4746 and 227 and 129 for R5297.
P values were calculated for lengths measured at 0 min and 30 min for non-competent R1501 (approximate $P = 0.4794$), competent R1501 (approximate $P<0.0001$), non-competent R4599 (exact $P = 0.8928$), competent R4599 (exact $P<0.0001$), non-competent R4743 (approximate $P = 0.188$), competent R4743 (approximate $P<0.0001$), non-competent R4728 (exact $P = 0.1093$), competent R4728 (exact $P = 0.0001$), non-competent R4867 (approximate $P = 0.9873$), competent R4867 (approximate $P<0.0001$), non-competent R4869 (approximate $P = 0.0.0696$), competent R4869 (exact $P = 0.0005$), non-competent R4746 (approximate $P = 0.0684$), competent R4746 (approximate $P<0.0001$), non-competent R5297 (approximate $P = 0.2331$), and competent R5297 (approximate $P<0.0001$).
Source data are provided as a Source data file.

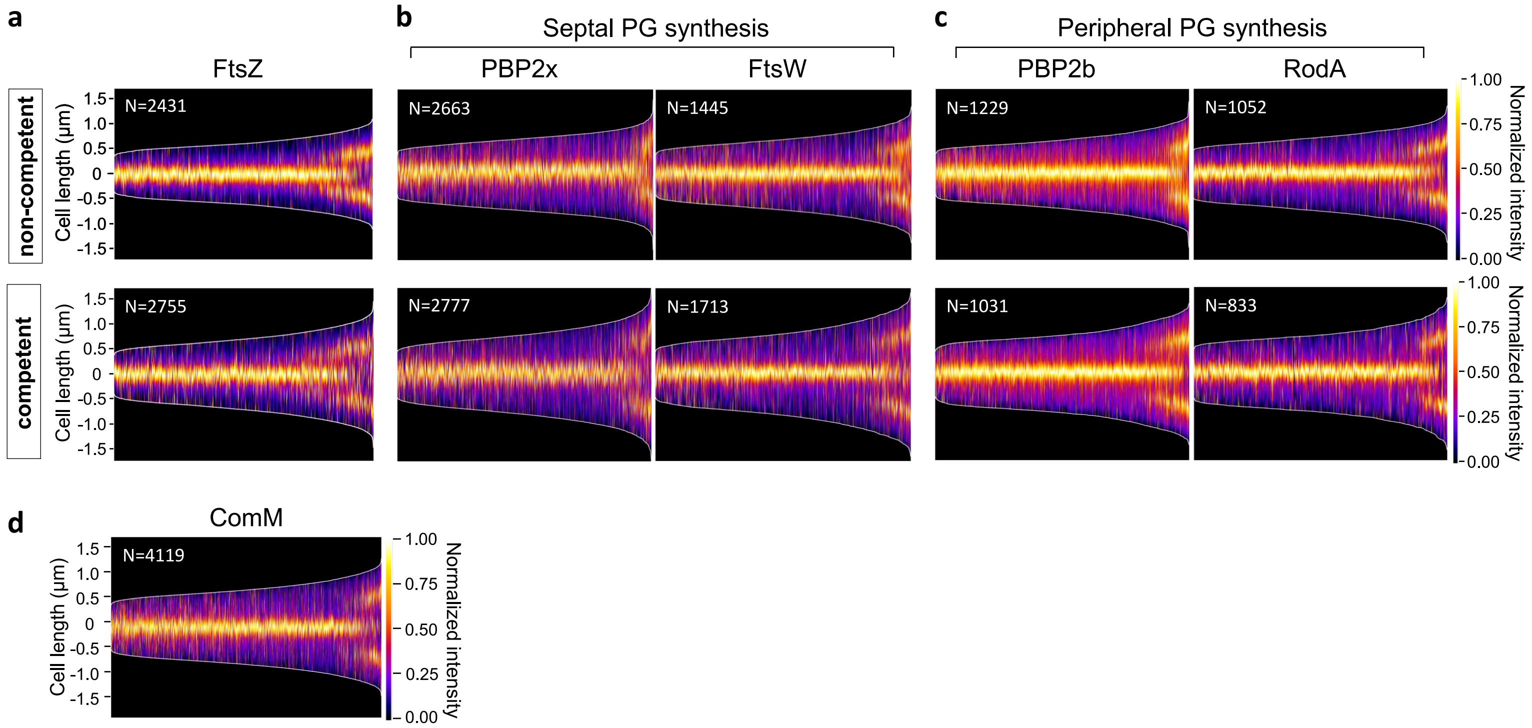

Supplementary Figure 6: Deployment of Divisome proteins in competent and non-competent cells.
Cells were grown in C+Y medium to early exponential phase and competence was induced, or not, by CSP addition for 30 min before imaging. The data are representative of three independent biological replicates. Cells were ordered by cell length and represented by a heatmap of the fluorescence signal. The number of cells analyzed is indicated (N).
a-c Demographs showing the localization signal of FtsZ-mNeonGreen (strain R4599) (a), of proteins of the septal PG synthesis machinery: mNeongreen-PBP2x (R4743) and FtsW-mNeonGreen (R4728) (b), and of proteins of the peripheral PG synthesis machinery: GFP-PBP2b (strain WT gfp-pbp2b) and RodA-mNeonGreen (R4867) (c), in non-competent (top panels) and competent cells (bottom panels).
d Demograph showing the localization signal of mNeonGreen-ComM (strain R4601) in competent cells.

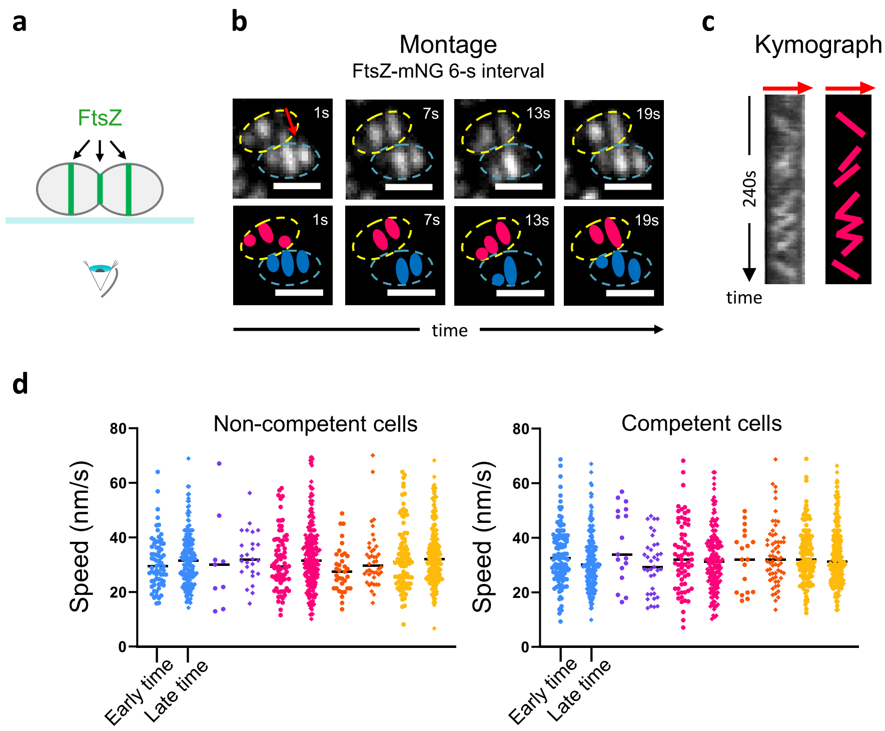

Supplementary Figure 7. Dynamics of FtsZ remains unchanged at all stages of the cell cycle in competent cells

a Schematics of the method to observe FtsZ-mNeonGreen in S. pneumoniae cells lying horizontally, with the division plane orthogonal to the coverslip (related to Fig. 2a and Supplementary Fig. 3).

b *Top panels*, Montage of TIRFM images at 6 s intervals showing FtsZ-mNeonGreen (strain R4599). The red arrow indicates the trajectory (voluntarily slightly shifted to the right to allow the visualization of the FtsZ signal) extracted for the kymograph analysis shown in c. *Bottom panels*, Corresponding cartoon representation of the FtsZ filaments in the two cells shown (red and blue dots). The contours of the two cells are represented in yellow and turquoise dotted lines. Scale bars, 1 µm.

c *Left*, Kymograph from 1 to 240 s obtained from the trajectory shown in b. *Right*, Cartoon representation of the kymograph.

d Speed of FtsZ-mNeonGreen (strain R4599) in different Z-ring types as represented in Fig. 2b in non-competent and competent cells at different times after competence induction (Early time, 10-25 min after CSP addition; Late time, 30-55 min after CSP addition). A total of 1021 and 1038 trajectories were analyzed over two independent replicates in non-competent cells and competent cells, respectively. Two-sided one-way ANOVA statistical analysis indicate no significant differences between conditions (ns, exact P = 0.2792 for non-competent cells and P = 0.2333 for competent cells).

Source data are provided as a Source data file.

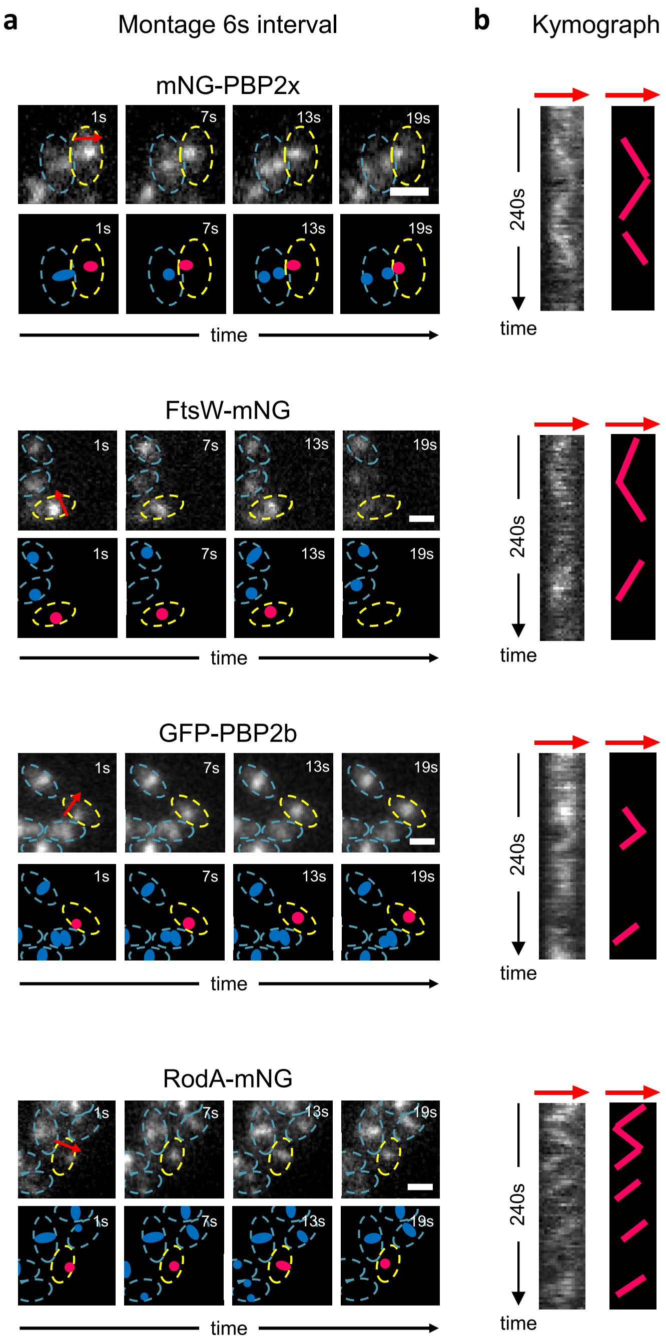

Supplementary Figure 8. Kymograph analyzes indicate that the speed of PBP2x and FtsW is reduced during competence compared to the speed of PBP2b and RodA.
Representative data are shown from two independent biological replicates.
a *Upper parts*: Montage of TIRFM images at 6 s intervals showing fusions of mNeonGreen or GFP with PBP2x (strain R4743), FtsW (strain R4728), PBP2b (strain WT gfp-pbp2b) and RodA (strain R4867). Red arrows indicate the trajectory extracted for kymograph analyses (voluntarily slightly shifted to allow the visualization of the fluorescent patches). *Lower part*: Cartoon representation of the fluorescent images (red and blue dots represent distinct fluorescent patches). The cell contour is represented in yellow and blue dotted lines. Scale bars, 1 µm.
b Kymographs from 1 to 240 s obtained from the trajectories shown in panel a (*Left*), and cartoon representations (*Right*). The slope of the fluorescent patches represented as red dots in panel a are shown.

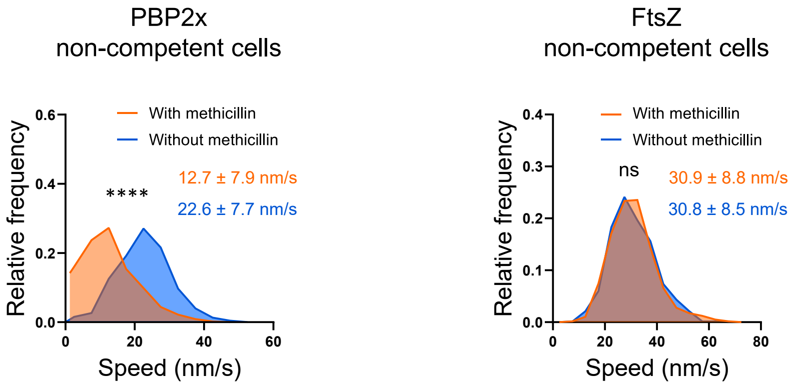

Supplementary Figure 9. Methicillin arrests the dynamics of PBP2x but not of FtsZ.
Distribution of speed of mNeonGreen-PBP2x (strain R4743) in a population of non-competent cells treated (455 trajectories) or not (454 trajectories), with methicillin and distribution of speed of FtsZ-mNeonGreen (R4599) in non-competent cells treated (578 trajectories) or not (667 trajectories) with methicillin. The average speed ± sd for each condition is indicated. Pairwise comparisons were done with a nonparametric two-sided Mann-Whitney test; ****, $P < 0.0001$; ns, $P > 0.05$. Approximate P values were calculated for strain R4743 (P<0.0001) and strain R4599 (P = 0.9028). Source data obtained from two independent biological replicates are provided as a Source data file.

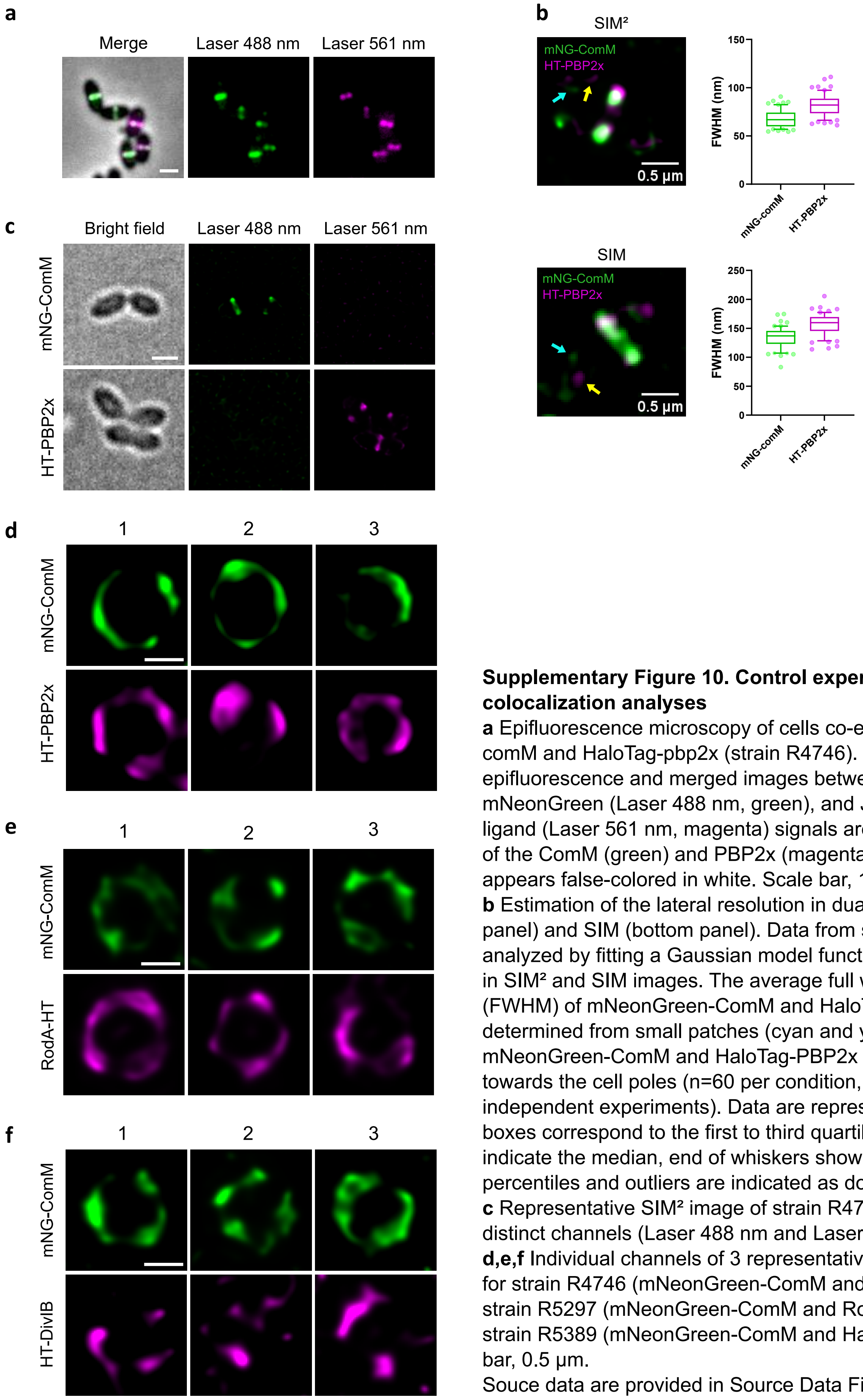

Supplementary Figure 10. Control experiments for colocalization analyses

a Epifluorescence microscopy of cells co-expressing mNeonGreen-comM and HaloTag-pbp2x (strain R4746). Representative epifluorescence and merged images between bright field (gray), mNeonGreen (Laser 488 nm, green), and Janelia fluor HaloTag ligand (Laser 561 nm, magenta) signals are shown. Superimposition of the ComM (green) and PBP2x (magenta) fluorescence signals appears false-colored in white. Scale bar, 1 µm.

b Estimation of the lateral resolution in dual color SIM² images (top panel) and SIM (bottom panel). Data from strains R4746 were analyzed by fitting a Gaussian model function to the intensity profile in SIM² and SIM images. The average full width at half maximum (FWHM) of mNeonGreen-ComM and HaloTag-PBP2x were determined from small patches (cyan and yellow arrows for mNeonGreen-ComM and HaloTag-PBP2x respectively) visible towards the cell poles (n=60 per condition, analyzed over two independent experiments). Data are represented as box plots where boxes correspond to the first to third quartiles, lines inside the boxes indicate the median, end of whiskers show the 10th and 90th percentiles and outliers are indicated as dots.

c Representative SIM² image of strain R4746 in bright field and two distinct channels (Laser 488 nm and Laser 561 nm).

d,e,f Individual channels of 3 representative rings imaged by SIM² for strain R4746 (mNeonGreen-ComM and HaloTag-PBP2x) (**d**), strain R5297 (mNeonGreen-ComM and RodA-HaloTag) (**e**), and strain R5389 (mNeonGreen-ComM and HaloTag-DivIB) (**f**). Scale bar, 0.5 µm.

Souce data are provided in Source Data File.

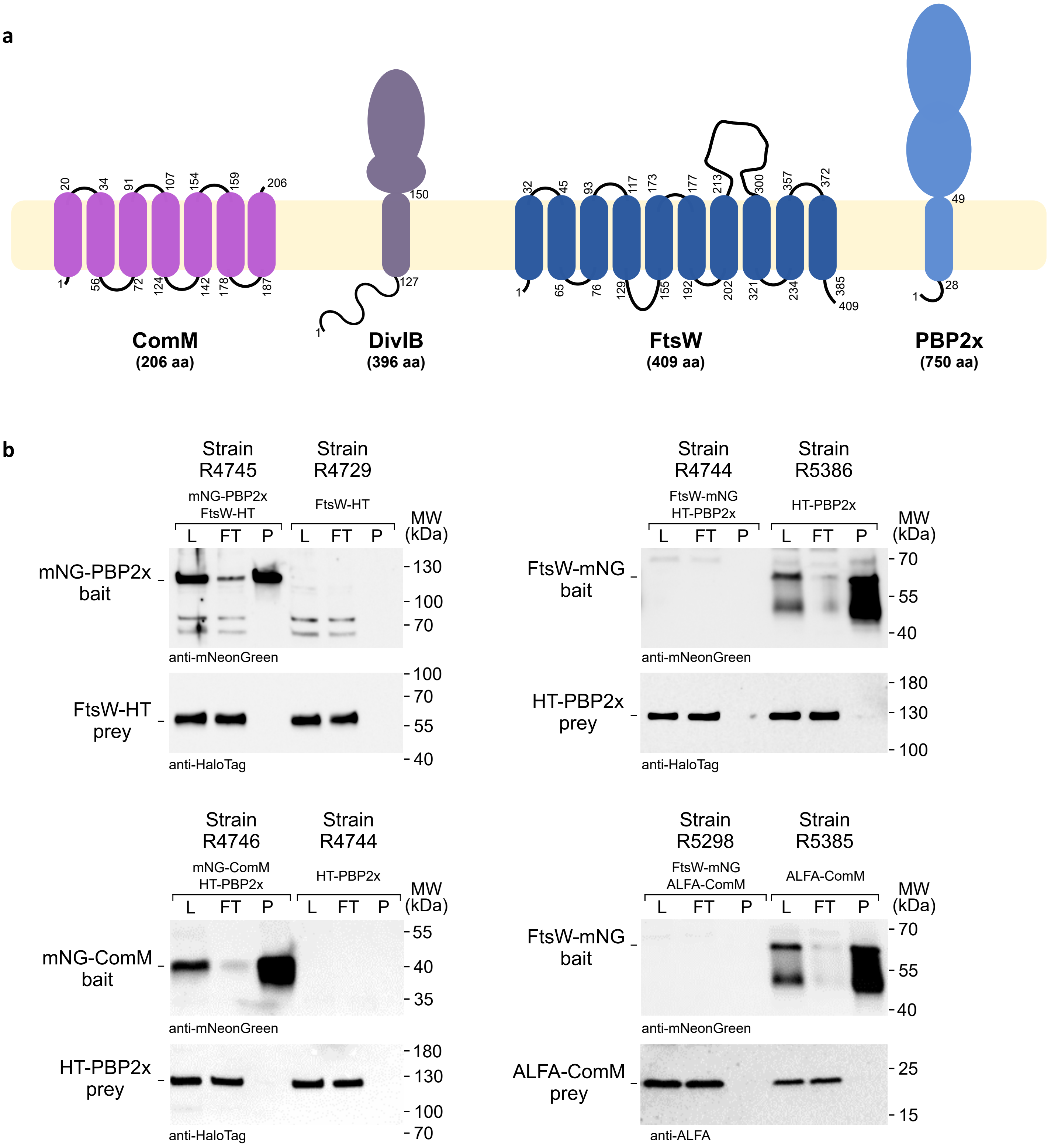

Supplementary Figure 11. Western blot analysis of co-immunoprecipitation assays
a Schematic representation of the membrane proteins ComM, DivIB, FtsW and PBP2x. Models are based on DeepTMHMM prediction.
b Immunoblots from co-immunoprecipitation assays of strains R4745 (mNeonGreen-PBP2x, FtsW-HaloTag), R4729 (FtsW-HaloTag), R4744 (HaloTag-PBP2x), R5386 (FtsW-mNeonGreen, HaloTag-PBP2x), R4746 (mNeonGreen-ComM, HaloTag-PBP2x), R5298 (ALFA-ComM) and R5385 (FtsW-mNeonGreen, ALFA-ComM). Immunoprecipitations using mNeonGreen traps were performed on detergent-solubilized membrane fractions derived from pneumococcal cells 15 min after competence induction. Detergent solubilized membranes prior to immunoprecipitation (L, load), the supernatants after immunoprecipitation (FT, flow-through), and the immunoprecipitations (IP) were subjected to immunoblot analysis using anti-mNeonGreen ,anti-HaloTag and anti-ALFA antibodies. Equivalent amounts of the load and supernatant (~3 ml equivalent culture) and approximately five (~14 ml equivalent culture for anti-mNeongreen immunoblot analysis) and six (~17ml equivalent culture for anti-HaloTag and anti-ALFA immunoblot analysis) times more of the IP were analyzed.
Uncropped blots are provided in Source Data File.

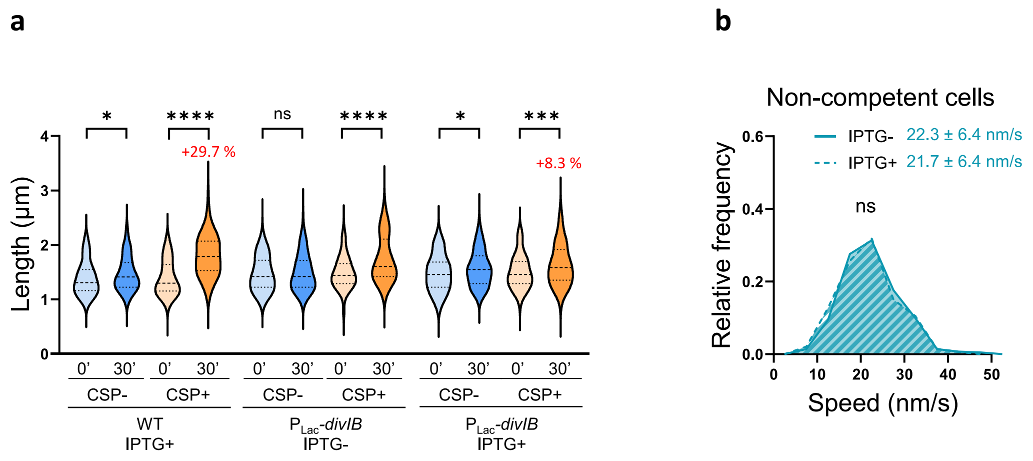

Supplementary Figure 12. Overproduction of DivIB partially rescues the ComM-dependent cell elongation phenotype of competent cells.

a Cell length distributions measured on phase contrast microscopy images using > 100 cells for each strain. Cells from strains R1501 and R5224 (P_{Lac}-divIB) overexpressing (IPTG+, 486 trajectories) or not (IPTG-, 431 trajectories) DivIB were imaged during 1 hour after incubation with or without CSP. Dimensions were measured 0 min and 30 min post-CSP addition. Data are presented as violin plots where the top and bottom dashed lines correspond to the 25th and 75th percentiles, and the middle dashed line corresponds to the median. Pairwise comparisons were done with a nonparametric two-sided Mann-Whitney test. *P* values are displayed as follows: ****, $P < 0.0001$; ***, $0.0001 < P < 0.001$; *, $0.01 < P < 0.05$; ns, $P > 0.05$. Approximate *P* values were calculated for lengths measured at 0 min and 30 min for non-competent R1501 ($P = 0.0175$), competent R1501 ($P<0.0001$), non-competent R5224 ($P = 0.6557$), competent R5224 ($P<0.0001$), non-competent R5224 treated with IPTG ($P = 0.0415$), and competent R5224 treated with IPTG ($P = 0.0009$). For the control condition and the strain overproducing DivIB, the average increase in length in competent cells is displayed in red.

b Distribution of speed of mNeonGreen-PBP2x patches in non competent cells overexpressing (IPTG+) or not (IPTG-) DivIB (strain R5225). Average speed ± sd for each condition is indicated. For each condition, a minimum of 250 trajectories were analyzed. There is no significant difference between the two values according to the two-sided Mann-Whitney nonparametric test (approximate $P = 0.2161$).

Source data are provided as aSource data file.

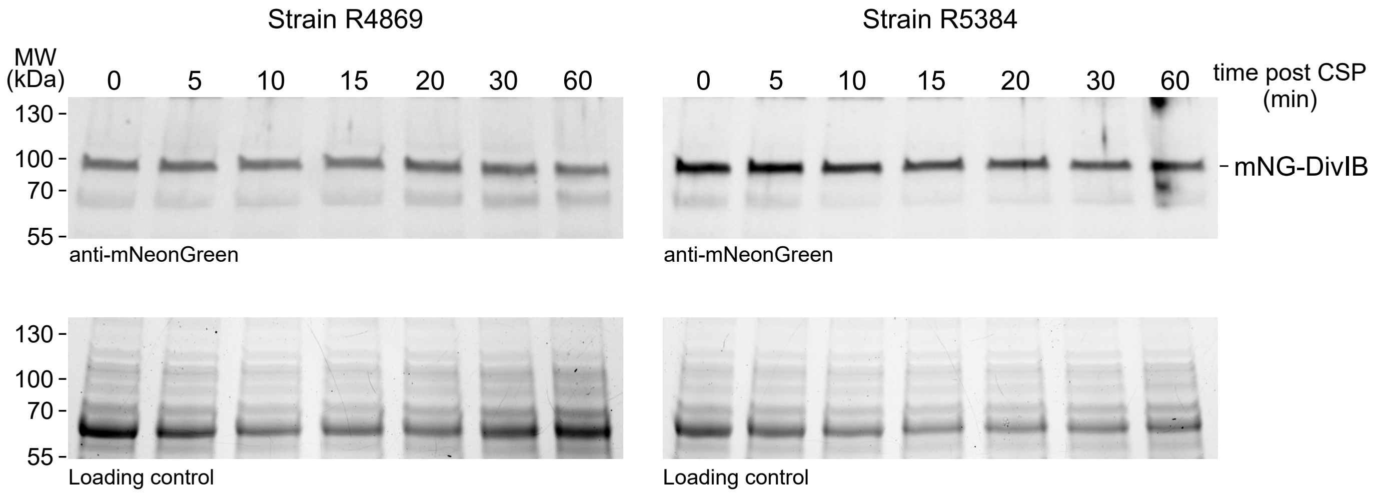

Supplementary Figure 13. DivIB remains stable during the course of competence
Western blot detection of mNeonGreen-DivIB at different times after competence induction (post-CSP addition). Strains analyzed: R4869 (mNeonGreen-DivIB) and R5384 (mNeonGreen-DivIB, Δ*comM*). Whole cell extracts were analyzed with anti-mNeonGreen antibodies. Stain free images were used as loading controls. Data representative of two independent experiments.
Uncropped blots are provided in Source Data File.

TCAGGATCTGGTGGAGAAGCAGCAGCTAAAGCTGGAGCTGAAATTGGTACTGGTTTTC
CATTTGATCCACATTATGTTGAAGTTTTGGGTGAACGTATGCATTATGTTGATGTTGGTC
CACGTGATGGTACTCCAGTTTTGTTTTTGCATGGTAATCCAACAAGTTCTTATGTTTGGC
GAAATATTATTCCACATGTTGCTCCAACACATCGTTGTATTGCTCCAGATCTTATTGGTAT
GGGTAAATCTGATAAACCAGATTTGGGTTATTTTTTTGATGATCATGTTCGTTTTATGGAT
GCTTTTATTGAAGCTTTGGGTTTGGAAGAAGTTGTTTTGGTTATTCATGATTGGGGTTCT
GCTTTGGGTTTTCATTGGGCTAAACGTAATCCAGAACGTGTTAAAGGTATTGCTTTTATG
GAATTTATTCGTCCAATTCCAACATGGGATGAATGGCCAGAATTTGCTCGTGAAACATTT
CAAGCTTTTCGTACAACAGATGTTGGTCGTAAATTGATTATTGATCAAAATGTTTTTATTG
AAGGTACATTGCCAATGGGTGTTGTTCGTCCATTGACAGAAGTTGAAATGGATCATTATC
GTGAACCATTTTTGAATCCAGTTGATCGTGAACCATTGTGGCGTTTTCCAAATGAATTGC
CAATCGCTGGTGAACCAGCTAATATTGTTGCTTTGGTTGAAGAATATATGGATTGGTTGC
ATCAATCTCCAGTTCCAAAATTGTTGTTTTGGGGTACACCAGGTGTTTTGATTCCACCAG
CTGAAGCTGCTCGTTTGGCTAAATCTTTGCCAAATTGTAAAGCTGTTGATATTGGTCCAG
GTTTGAATTTGTTGCAAGAAGATAATCCAGATTTGATTGGTTCTGAAATTGCTCGTTGGT
TGTCTACATTGGAAATCTCTGGTTTGGAAGGATCAGGACAAGGACCAGGAAGTGGTCAA
GGTTCAGGT

Supplementary Figure 14. Sequence of the DNA fragment containing the HAloTag sequence. The DNA fragment carries the HaloTag sequence codon optimized for *S. pneumoniae* published with the Winkler laboratory[4] with a few modifications indicated in red. It also contains the L5 (bold)[5] and L6 (underlined)[4] linkers at the 5' and 3' extremities, respectively.

Supplementary References

1. Dagkessamanskaia, A. *et al.* Interconnection of competence, stress and CiaR regulons in *Streptococcus pneumoniae*: competence triggers stationary phase autolysis of ciaR mutant cells. *Molecular Microbiology* **51**, 1071–1086 (2004).

2. Johnston, C. H. G. *et al.* The RecA-directed recombination pathway of natural transformation initiates at chromosomal replication forks in the pneumococcus. *Proc Natl Acad Sci U S A* **120**, e2213867120 (2023).

3. Fleurie, A. *et al.* Interplay of the serine/threonine-kinase StkP and the paralogs DivIVA and GpsB in pneumococcal cell elongation and division. *PLoS Genet* **10**, e1004275 (2014).

4. Perez, A. J. et al. Movement dynamics of divisome proteins and PBP2x:FtsW in cells of Streptococcus pneumoniae. Proc Natl Acad Sci USA 116, 3211–3220 (2019).

5. van Raaphorst, R., Kjos, M. & Veening, J.-W. Chromosome segregation drives division site selection in Streptococcus pneumoniae. Proc Natl Acad Sci USA 114, E5959–E5968 (2017).
