# Supplementary material for: Transient inhibition of cell division in competent pneumococcal cells results from deceleration of the septal peptidoglycan complex
Source: Nat Commun. 2025 Jul 1;16:5666. doi: 10.1038/s41467-025-60600-z (PMC12214887; doi:10.1038/s41467-025-60600-z)
Supplement: Supplementary file 2 — Description of Additional Supplementary Files [file 41467_2025_60600_MOESM2_ESM.pdf]

Description of Additional Supplementary Materials:

Supplementary Movie 1: ComM forms dynamic patches moving around the cell circumference. Cell expressing mNeonGreen-ComM (strain R4601) imaged in a vertical chamber. Cells were grown in C+Y medium to early exponential phase and induced to develop competence by CSP addition before imaging. 1 frame every 5 s.

Supplementary Movie 2: ComM forms dynamic patches moving around the cell circumference. Cells expressing mNeonGreen-ComM (strain R4601) horizontally positioned on the slide. Cells were grown in C+Y medium to early exponential phase and induced to develop competence by CSP addition before imaging. 1 frame every 3 s. The montage comprises three panels, arranged from left to right, with the image time sequence, the average projection, and the kymograph generated on the particle targeted by an arrow. A mobile cursor traverses the kymograph, thereby establishing a correlation between the movement of a protein on the film and its representation in the kymograph.

Supplementary Movie 3: FtsZ shows bidirectional movements within the Z-rings. Cells expressing FtsZmNeonGreen (strain R4599) grown in C+Y medium to early exponential phase. 1 frame every 3 s. The montage comprises three panels, arranged from left to right, with the image time sequence, the average projection, and the kymograph generated on the particle targeted by an arrow. A mobile cursor traverses the kymograph, thereby establishing a correlation between the movement of a protein on the film and its representation in the kymograph.

Supplementary Movie 4: PBP2x exhibits bidirectional processive movement at the site of cell division. Cells expressing mNeonGreen-PBP2x (strain R4743) grown in C+Y medium to early exponential phase. 1 frame every 3 s. The montage comprises three panels, arranged from left to right, with the image time sequence, the average projection, and the kymograph generated on the particle targeted by an arrow. A mobile cursor traverses the kymograph, thereby establishing a correlation between the movement of a protein on the film and its representation in the kymograph.

Supplementary Movie 5: FtsW exhibits bidirectional processive movements at the site of cell division. Cells expressing FtsW-mNeonGreen (strain R4728) grown in C+Y medium to early exponential phase. 1 frame every 3 s. The montage comprises three panels, arranged from left to right, with the image time sequence, the average projection, and the kymograph generated on the particle targeted by an arrow. A mobile cursor traverses the kymograph, thereby establishing a correlation between the movement of a protein on the film and its representation in the kymograph.

Supplementary Movie 6: PBP2b exhibits bidirectional processive movements at the site of cell division. Cells expressing GFP-PBP2b (strain WT gfp-pbp2b) grown in C+Y medium to early exponential phase. 1 frame every 3 s. The montage comprises three panels, arranged from left to right, with the image time sequence, the average projection, and the kymograph generated on the particle targeted by an arrow. A mobile cursor traverses the kymograph, thereby establishing a correlation between the movement of a protein on the film and its representation in the kymograph.

Supplementary Movie 7: RodA exhibits bidirectional processive movements at the site of cell division. Cells expressing RodA-mNeonGreen (strain R4867) grown in C+Y medium to early exponential phase. 1 frame every 3 s. The montage comprises three panels, arranged from left to right, with the image time sequence, the average projection, and the kymograph generated on the particle targeted by an arrow. A mobile cursor traverses the kymograph, thereby establishing a correlation between the movement of a protein on the film and its representation in the kymograph.

Supplementary Movie 8: DivIB exhibits bidirectional processive movements at the site of cell division. Cell expressing mNeonGreen-DivIB (strain R4869) grown in C+Y medium to early exponential phase. 1 frame every 3 s. The montage comprises three panels, arranged from left to right, with the image time sequence, the average projection, and the kymograph generated on the particle targeted by an arrow. A mobile cursor traverses the kymograph, thereby establishing a correlation between the movement of a protein on the film and its representation in the kymograph.
