# Supplementary material for: Transient inhibition of cell division in competent pneumococcal cells results from deceleration of the septal peptidoglycan complex
Source: Nat Commun. 2025 Jul 1;16:5666. doi: 10.1038/s41467-025-60600-z (PMC12214887; doi:10.1038/s41467-025-60600-z)
Supplement: Supplementary file 11 — Reporting Summary [file 41467_2025_60600_MOESM11_ESM.pdf]

nature portfolio

Corresponding author(s): Nathalie Campo and Rut Carballido-Lopez

Last updated by author(s): May 13, 2025

Reporting Summary

Nature Portfolio wishes to improve the reproducibility of the work that we publish. This form provides structure for consistency and transparency in reporting. For further information on Nature Portfolio policies, see our Editorial Policies and the Editorial Policy Checklist.

Statistics

For all statistical analyses, confirm that the following items are present in the figure legend, table legend, main text, or Methods section.

n/a	Confirmed	
☐	☒	The exact sample size (*n*) for each experimental group/condition, given as a discrete number and unit of measurement
☐	☒	A statement on whether measurements were taken from distinct samples or whether the same sample was measured repeatedly
☐	☒	The statistical test(s) used AND whether they are one- or two-sided<br>*Only common tests should be described solely by name; describe more complex techniques in the Methods section.*
☒	☐	A description of all covariates tested
☒	☐	A description of any assumptions or corrections, such as tests of normality and adjustment for multiple comparisons
☐	☒	A full description of the statistical parameters including central tendency (e.g. means) or other basic estimates (e.g. regression coefficient) AND variation (e.g. standard deviation) or associated estimates of uncertainty (e.g. confidence intervals)
☐	☒	For null hypothesis testing, the test statistic (e.g. *F*, *t*, *r*) with confidence intervals, effect sizes, degrees of freedom and *P* value noted<br>*Give P values as exact values whenever suitable.*
☒	☐	For Bayesian analysis, information on the choice of priors and Markov chain Monte Carlo settings
☒	☐	For hierarchical and complex designs, identification of the appropriate level for tests and full reporting of outcomes
☐	☒	Estimates of effect sizes (e.g. Cohen's *d*, Pearson's *r*), indicating how they were calculated

Our web collection on statistics for biologists contains articles on many of the points above.

Software and code

Policy information about availability of computer code

Data collection	Data were collected with the following software tools:<br>• Image Lab 5.2 software (Bio-Rad) for image acquisition and analysis of western blots;<br>• SkanIt Software 7.0.2 (Thermo Scientific) for measurement of luminescence in split-luciferase complementation assays;<br>• MetaMorph software v7.10.5.476 (Moleular Devices) for Epifluorescence microscopy;<br>• ZEN 2012 SP2 Black (Carl Zeiss Microscopy, v11.0.4.190), for TIRF and HILO imaging;<br>• ZEN 3.0 SR FP2 Black (Carl Zeiss Microscopy, v16.0.20.306), for Lattice SIM image acquisition.
Data analysis	Collected data were analyzed with the following software tools:<br>• GraphPad Prism 10 (GraphPad Software, LLC), for graphical representation and statistical analysis;<br>• Fiji (v2.14.0 / 1.54f; https://imagej.net/software/fiji/), for microscopy image preparation, data quantification, and speed measurements of circumferentially moving proteins in vertically oriented cells (https://github.com/CyrilleBillaudeau/CircleKymolJ);<br>• MATLAB (Mathworks, R2018b) for quantification of Speeds of directionally moving proteins in horizontally oriented cells; ((https://github.com/CyrilleBillaudeau/Kymo_Analyser_MultiChannel);<br>• Microbe J (version 5.13n) for generation demographs and heat maps;<br>• Zen Software (Zeiss, black edition) for Lattice SIM image reconstruction;<br>• Python (v3.10) with open-source package Proced-deepseg (https://github.com/aurelien-barbotin/proced-deepseg), for single cell doubling time and morphologic quantification.

For manuscripts utilizing custom algorithms or software that are central to the research but not yet described in published literature, software must be made available to editors and reviewers. We strongly encourage code deposition in a community repository (e.g. GitHub). See the Nature Portfolio guidelines for submitting code & software for further information.

Data

Policy information about availability of data

All manuscripts must include a data availability statement. This statement should provide the following information, where applicable:

- Accession codes, unique identifiers, or web links for publicly available datasets
- A description of any restrictions on data availability
- For clinical datasets or third party data, please ensure that the statement adheres to our policy

Source data are provided with this paper. Research data (fluorescent images and all movies used to measure speeds of single particles including 10-15 examples of kymographs per condition) have been deposited in the public repository platform Zenodo (https://doi.org/10.5281/zenodo.15394703). All relevant data are available in this article and its Supplementary information files.
The analysis code used in this study for single cell doubling time and morphologic quantification was written in Python (v3.10) and is deposited on GitHub at https://github.com/aurelien-barbotin/proced-deepseg.
The analysis codes used in this study for speed measurements of circumferentially moving proteins were written in MATLAB language for horizontally oriented cells and in Fiji/ImageJ scripts for vertically oriented cells and are deposited on GitHub at https://github.com/CyrilleBillaudeau/Kymo_Analyser_MultiChannel and https://github.com/CyrilleBillaudeau/CircleKymoIJ, respectively.

Research involving human participants, their data, or biological material

Policy information about studies with human participants or human data. See also policy information about sex, gender (identity/presentation), and sexual orientation and race, ethnicity and racism.

Reporting on sex and gender	N/A
Reporting on race, ethnicity, or other socially relevant groupings	N/A
Population characteristics	N/A
Recruitment	N/A
Ethics oversight	N/A

Note that full information on the approval of the study protocol must also be provided in the manuscript.

Field-specific reporting

Please select the one below that is the best fit for your research. If you are not sure, read the appropriate sections before making your selection.

[x] Life sciences [ ] Behavioural & social sciences [ ] Ecological, evolutionary & environmental sciences

For a reference copy of the document with all sections, see nature.com/documents/nr-reporting-summary-flat.pdf

Life sciences study design

All studies must disclose on these points even when the disclosure is negative.

Sample size	No sample size calculation was made. For all experiments, unless otherwise stated, data were acquired from three independently prepared biological replicates per condition/strain. Large number of cells were analysed in order to get close to a normal distribution. Sample size is specified in each figure legend.<br>For live cell imaging, single-cell doubling time and morphologic quantification, as well as demograph and heat-map analysis, at least three fields of microscopy view, each containing hundreds of cells were analysed over at least 2 independent experiments.<br>For SIM imaging, a minimum of 35 cells observed over 2 independent experiments were analyzed.<br>For speed measurement of directionally moving foci, a minimum of 145 (up to 1111) trajectories were analyzed over 3 independent experiments.
Data exclusions	No data were excluded, except for cells that were not maintained properly in vertical chambers and exhibited movements during image acquisition.
Replication	All attempts at replication were successful. For all experiments, unless otherwise stated, data were acquired from three independently prepared biological replicates per condition/strain. As results were representative of replicated studies, analyses represent the pooled data, the mean of multiple experiments or a representative experiment, as described in the Figure legends.
Randomization	N/A – all measurements and analyses were performed identically over all conditions.
Blinding	Blinding was not relevant as samples were processed identically over all conditions.

Reporting for specific materials, systems and methods

We require information from authors about some types of materials, experimental systems and methods used in many studies. Here, indicate whether each material, system or method listed is relevant to your study. If you are not sure if a list item applies to your research, read the appropriate section before selecting a response.

Materials & experimental systems

n/a	Involved in the study
☐	☒ Antibodies
☒	☐ Eukaryotic cell lines
☒	☐ Palaeontology and archaeology
☒	☐ Animals and other organisms
☒	☐ Clinical data
☒	☐ Dual use research of concern
☒	☐ Plants

Methods

n/a	Involved in the study
☒	☐ ChIP-seq
☒	☐ Flow cytometry
☒	☐ MRI-based neuroimaging

Antibodies

Antibodies used	Primary antibodies:<br>• anti-HaloTag (G921A, Promega), diluted 1:5,000;<br>• anti-ALFA (N1581, NanoTag Biotechnologies), diluted 1:10,000;<br>• anti-mNeonGreen (Eurogentec, custom made), diluted 1:10,000.<br><br>Secondary antibodies:<br>• peroxidase-conjugated goat anti-rabbit immunoglobulin G (A0545, Sigma) diluted 1:10,000.
Validation	• anti-HaloTag (G921A, Promega): https://france.promega.com/products/protein-detection/primary-and-secondary-antibodies/anti-halotag-monoclonal-antibody/?catNum=G9211<br>• anti-ALFA (N1581, NanoTag Biotechnologies): blob:https://nano-tag.com/2afa284a-470b-49df-9fb7-1411eff689a2<br>• peroxidase-conjugated goat anti-rabbit immunoglobulin G (A0545, Sigma): https://www.sigmaaldrich.com/deepweb/assets/sigmaaldrich/product/documents/392/007/a0545dat-mk.pdf<br>• Supplementary Figure 1a in this study shows that custom made anti-mNeonGreen (Eurogentec) specifically detects mNeonGreen protein fusions.

Plants

Seed stocks	N/A
Novel plant genotypes	N/A
Authentication	N/A
